# Association of Chronic Low-grade Inflammation With Risk of Alzheimer Disease in *ApoE4* Carriers

**DOI:** 10.1001/jamanetworkopen.2018.3597

**Published:** 2018-10-19

**Authors:** Qiushan Tao, Ting Fang Alvin Ang, Charles DeCarli, Sanford H. Auerbach, Sheral Devine, Thor D. Stein, Xiaoling Zhang, Joseph Massaro, Rhoda Au, Wei Qiao Qiu

**Affiliations:** 1 Department of Pharmacology and Experimental Therapeutics, Boston University School of Medicine, Boston, Massachusetts; 2 Department of Anatomy and Neurobiology, Boston University School of Medicine, Boston, Massachusetts; 3 Department of Epidemiology, Boston University School of Public Health, Boston, Massachusetts; 4 Alzheimer’s Disease Center, University of California Davis Medical Center, Sacramento; 5 Department of Neurology, Boston University School of Medicine, Boston, Massachusetts; 6 Framingham Heart Study, Boston University School of Medicine, Boston, Massachusetts; 7 Department of Psychiatry, Boston University School of Medicine, Boston, Massachusetts; 8 Department of Pathology and Laboratory Medicine, Boston University School of Medicine, Boston, Massachusetts; 9 Department of Pathology, Veterans Affairs Boston Healthcare System, Boston, Massachusetts; 10 Alzheimer’s Disease Center, Boston University School of Medicine, Boston, Massachusetts; 11 Department of Medicine, Boston University School of Medicine, Boston, Massachusetts; 12 Department of Biostatistics, Boston University School of Public Health, Boston, Massachusetts

## Abstract

**Importance:**

The association between peripheral inflammatory biomarkers and Alzheimer disease (AD) is not consistent in the literature. It is possible that chronic inflammation, rather than 1 episode of inflammation, interacts with genetic vulnerability to increase the risk for AD.

**Objective:**

To study the interaction between the apolipoprotein E (*ApoE*) genotype and chronic low-grade inflammation and its association with the incidence of AD.

**Design, Setting, and Participants:**

In this cohort study, data from 2656 members of the Framingham Heart Study offspring cohort (Generation 2; August 13, 1971-November 27, 2017) were evaluated, including longitudinal measures of serum C-reactive protein (CRP), diagnoses of incident dementia including AD, and brain volume. Chronic low-grade inflammation was defined as having CRP at a high cutoff level at a minimum of 2 time points. Statistical analysis was performed from December 1, 1979, to December 31, 2015.

**Main Outcomes and Measures:**

Development of AD and brain volumes.

**Results:**

Of the 3130 eligible participants, 2656 (84.9%; 1227 men and 1429 women; mean [SD] age at last CRP measurement, 61.6 [9.5] years) with both *ApoE* status and longitudinal CRP measurements were included in this study analysis. Median (interquartile range) CRP levels increased with mean (SD) age (43.3 [9.6] years, 0.95 mg/L [0.40-2.35 mg/L] vs 59.1 [9.6] years, 2.04 mg/L [0.93-4.75 mg/L] vs 61.6 [9.5] years, 2.21 mg/L [1.05-5.12 mg/L]; *P* < .001), but less so among those with *ApoE4* alleles, followed by *ApoE3* then *ApoE2* genotypes. During the 17 years of follow-up, 194 individuals (7.3%) developed dementia, 152 (78.4%) of whom had AD. *ApoE4* coupled with chronic low-grade inflammation, defined as a CRP level of 8 mg/L or higher, was associated with an increased risk of AD, especially in the absence of cardiovascular diseases (hazard ratio, 6.63; 95% CI, 1.80-24.50; *P* = .005), as well as an increased risk of earlier disease onset compared with *ApoE4* carriers without chronic inflammation (hazard ratio, 3.52; 95% CI, 1.27-9.75; *P* = .009). This phenomenon was not observed among *ApoE3* and *ApoE2* carriers with chronic low-grade inflammation. Finally, a subset of 1761 individuals (66.3%) underwent brain magnetic resonance imaging, and the interaction between *ApoE4* and chronic low-grade inflammation was associated with brain atrophy in the temporal lobe (β = –0.88, SE = 0.22; *P* < .001) and hippocampus (β = –0.04, SE = 0.01; *P* = .005), after adjusting for confounders.

**Conclusions and Relevance:**

In this study, peripheral chronic low-grade inflammation in participants with *ApoE4* was associated with shortened latency for onset of AD. Rigorously treating chronic systemic inflammation based on genetic risk could be effective for the prevention and intervention of AD.

## Key Points

**Question:**

Is interaction of chronic low-grade inflammation and the apolipoprotein E genotype associated with development of Alzheimer disease?

**Findings:**

In this population-based cohort study, chronic low-grade inflammation, assessed by longitudinal C-reactive protein measurements, was associated with an increased risk of Alzheimer disease only in *ApoE4* carriers.

**Meaning:**

Clinical follow-up and treatment of high levels of C-reactive protein may be beneficial for prevention of Alzheimer disease in *ApoE4* carriers.

## Teaser

This population-based cohort study uses data from the Framingham Heart Study offspring cohort to study the interaction between the apolipoprotein E genotype and chronic low-grade inflammation and its association with the incidence of Alzheimer disease.

## Introduction

The apolipoprotein E4 (*ApoE4* [OMIM 107741]) allele is the major genetic risk factor for late-onset Alzheimer disease (AD).^1^ However, not all *ApoE4* carriers develop AD, even among those older than 90 years.^2^ It is likely that a complex interaction of genetic vulnerabilities with environmental risk factors lead to AD and identifying such factors could be beneficial for the prevention of AD. One such interacting factor could be sustained or frequent systemic inflammations, as infections of the respiratory, gastrointestinal, and urinary tract systems are common in elderly individuals.

C-reactive protein (CRP) is an immune system response to toxins or injuries in systemic inflammation, while CRP levels increase with age.^3^ Although multiple AD-related genes are associated with the level of CRP,^4^ the association between blood CRP levels and risk of AD are not conclusive in the literature,^5,6,7^ with studies showing both low and high levels of CRP in patients with AD. Since AD is a chronic disease characterized by neurodegeneration in the brain, chronic low-grade inflammation, either sustained or frequently episodic, may be a risk factor for AD. However, most studies to date rely on one-time measurements of CRP and thus do not distinguish between a condition of acute inflammatory reaction followed by recovery and a condition of chronic inflammation without complete recovery or frequent episodic inflammation. Since preclinical studies suggest that CRP plays a role in *ApoE4* leading to AD,^4,8^ we thus hypothesized that the association of chronic elevated CRP levels with the risk of AD would be different across *ApoE* genotypes. This hypothesis prompted our study that includes longitudinal measures of high levels of CRP as a biomarker of chronic low-grade inflammation to determine the risk for development of AD.

The Framingham Heart Study is a large population-based, multigeneration cohort with long and intensive follow-up that includes multiple measurements of serum CRP taken during a 2-decade period.^9^ The purpose of this study was to determine if and how peripheral CRP levels are associated with the onset of AD in the context of *ApoE* genotypes. Included in this study is the Framingham Heart Study Generation 2 cohort enrolled in 1971, who had up to 3 CRP measurements between 1979 and 2001. Chronic low-grade inflammation was defined as meeting specified cutoff levels of plasma CRP in at least 2 measurements taken years apart. We examined the association between chronic low-grade inflammation, and risk of a diagnosis of dementia ,including AD, and brain volumes, stratified by *ApoE* genotype.

## Methods

### Study Design and Participants

The Framingham Heart Study is a single-site, community-based, prospective cohort study in Framingham, Massachusetts. The design and selection criteria of the Framingham Heart Study offspring cohort (Generation 2) have been previously described.^10^ The source population is 3130 participants, who were 20 years or older at the second health examination (1979-1983), had baseline CRP measured during that examination, and consented to use of their genetic information (ie, *ApoE* genotype). Excluded were 404 individuals who did not have another CRP measurement at the sixth (1995-1998) or seventh (1998-2001) health examinations and 46 individuals with the *ApoE2/4* genotype. In addition, 24 individuals with prevalent dementia at the time of each CRP measurement were excluded. Thus, the final study sample consisted of 2656 individuals (eFigure 1A in the Supplement), whose data were used for the primary analyses to examine the association between *ApoE*, CRP level, and risk of AD. Data on a subset of 1785 individuals, who also underwent brain magnetic resonance imaging (MRI) after the seventh examination (1999-2011) (eFigure 1B in the Supplement), were used for secondary analyses to examine the association between CRP, *ApoE*, and AD-related changes in brain structure. Cardiovascular disease status at examination 7 was used as a covariate and was represented as a dichotomous variable (yes or no), determined by the presence of the following conditions: myocardial infarction, angina pectoris, coronary insufficiency, congestive heart failure, and intermittent claudication. Written informed consent was obtained from all study participants and the study protocol was approved by the Institutional Review Board of Boston University Medical Campus. This study followed the Strengthening the Reporting of Observational Studies in Epidemiology (STROBE) reporting guideline. This study was monitored by a National Heart, Lung, and Blood Institute Observational Study Monitoring Board and followed their guidelines.

#### CRP Measurement

During the clinic visits, blood samples were drawn, under fasting condition, from the antecubital vein while participants were supine. Serum aliquots were frozen at –20°C after the initial phlebotomy and subsequently thawed for measurement of high-sensitivity CRP. Both CRP concentrations at examinations 2 and 6 were performed in the Framingham Heart Study laboratory using a previously described enzymatic immunoassay^11^ (Hemagen Diagnostics Inc). The CRP measurement at examination 7 was conducted by a Dade Behring BN100 nephelometer. The Pearson product moment correlation between both techniques was 0.98 and the CRP quartile assignment was identical.^9^

To define chronic low-grade inflammation, we used the following 2 criteria: (1) since a CRP level lower than 3 mg/L (to convert to nanomoles per liter, multiply by 9.524) is considered normal in a clinical setting, we defined low-grade inflammation as any CRP measurement above 3 mg/L and used different cutoff levels to indicate severity; and (2) chronic inflammatory status was defined as having at least 2 longitudinal CRP measurements above the stipulated cutoff levels. Participants with only 1 CRP measurement equal to or higher than 3 mg/L were not considered as having chronic low-grade inflammation.

#### Diagnoses of Dementia Including AD

Beginning in 1979, all Generation 2 participants have been followed up for incident dementia. The Mini-Mental State Examination (MMSE) was administered beginning at the fifth health examination (ie, 1991-1995) to monitor change in cognitive status. A performance drop in Mini-Mental State Examination score of 3 or more points from the immediately preceding examination or 5 or more points across all examinations would indicate a change in cognitive status that warranted review by a dementia diagnostic panel consisting of at least 1 neurologist and 1 neuropsychologist. Furthermore, from 1999 to 2005, all surviving Generation 2 participants were invited for an in-depth cognitive examination, which also screened for incident cognitive impairment that warranted review by the dementia diagnostic panel. Consensus diagnostic procedures have been previously described.^12^ Incidence of dementia, AD, and person-time accruement after the last longitudinal CRP measurement was used for analyses.

### Brain Measurements

The brain MRI was conducted beginning March 1999; only data acquired from brain MRI scans after the measurement of the last CRP (eg, examinations 6 or 7) were used. The brain MRI protocol has been reported in detail elsewhere.^13,14^ A Siemens 1-T MR machine (Siemens Medical) with a T2-weighted double spin-echo coronal imaging sequence was used. A central laboratory blinded to demographic and clinical information processed the digital information on brain images and quantified the brain data with a custom-written computer program operating on a UNIX, Solaris platform (Sun Microsystems).

The semiautomated segmentation protocol for quantifying total cranial volume, total cerebral brain volume, frontal lobar brain volume, parietal lobe brain volume, temporal lobe brain volume, and hippocampal volume has been described elsewhere,^15^ as have the interrater reliabilities for these methods. For segmentation of white matter hyperintensities from other brain tissues, the first and second images from T2 sequences were summed and a log-normal distribution was fitted to the summed data. A segmentation threshold for white matter hyperintensities was determined as 1 SD in pixel intensity greater than the mean of the fitted distribution of brain parenchyma. The units for the brain volumes, including total cerebral brain volume, frontal lobar brain volume, temporal lobe brain volume, hippocampal volume, and white matter hyperintensities, were computed as the percentage of total cranial volume. Each image set underwent rigorous quality control assessment that includes assessment of the original acquisition quality as well as the quality of the image processing. Moreover, each of the analysts was highly trained to maintain rigorous precision, with intraclass (analyst) coefficients above 90% for all analyses.

### Statistical Analysis

Statistical analysis was performed from December 1, 1979, to December 31, 2015. Analyses were performed using SAS software, version 9.3 (SAS Institute) and the R statistical environment (The R Foundation for Statistical Computing Platform 2017). We performed univariate analyses to describe baseline characteristics of the final sample population, stratified by *ApoE* genotype (*ApoE2-2/2* or *2/3*; *ApoE3-3/3*; *ApoE4-3/4* or *4/4*). Means and SDs were determined and analysis of variance tests were conducted on variables with normal distribution, Mann-Whitney tests were performed on variables with a skewed distribution using the median (interquartile range), and χ^2^ tests were used for categorical variables using number and percentage.

To establish the temporal sequence between chronic low-grade inflammation and the development of AD, we excluded individuals who did not have *ApoE* genotype measured, had an *ApoE2/4* genotype, did not have longitudinal CRP measurements, and/or had dementia at the time of or prior to the last measurement of CRP, leaving a total sample of 2656 (eFigure 1 in the Supplement). Cox proportional hazards regression models were used to examine chronic low-grade inflammation and the development of AD, dementia, or mortality after adjusting for age, sex, educational level, and cardiovascular disease; the interaction between *ApoE4* and chronic low-grade inflammation was examined as well. Kaplan-Meier survival analyses were performed to compare the onset of AD, dementia, or death among *ApoE* genotypes.

The secondary analysis focused on 1761 individuals who underwent a brain MRI after their last longitudinal CRP measurement (eFigure 1 in the Supplement). Multivariate linear regression was also used to study the association between chronic low-grade inflammation and total and regional brain volume measures, controlling for total brain volume, after adjusting for age (age groups, 20-29 years; 30-39 years, 40-49 years, and ≥50 years), sex, educational level, *ApoE4* status, and the time between the last CRP measurement and brain MRI scan. The interaction of the CRP status and *ApoE4* allele was also examined in these regression models for each brain measure. Given multiple testing and the need to minimize the rate of false positives, *P* values were adjusted using a conventional Bonferroni correction threshold of .005 in 2-sided *t* tests (eTable in the Supplement).

## Results

### Association of Median Levels of CRP With Age in the Context of *ApoE* Genotypes

The 2656 individuals who completed at least 2 CRP measurements and did not have dementia at the time of their last CRP measurement were a mean (SD) age of 61.4 (9.4) years (eFigure 1 in the Supplement). Participants were further categorized into the following 3 groups: *ApoE2* (n = 364), *ApoE3* (n = 1729), and *ApoE4* (n = 532) (Table 1). There were no differences in age, sex, educational levels, and prevalence of cardiovascular diseases among the *ApoE* subgroups.

**Table 1.** Demographic, Longitudinal CRP Measures, and Incident AD in *ApoE* Genotypes in the Framingham Heart Study Population

Characteristic	*ApoE2* Carriers (n = 367)	*ApoE3* Carriers (n = 1746)	*ApoE4* Carriers (n = 542)	*df*	*F* or χ^2^^a^	*P* Value^b^
Age after CRP measurements, y						
Mean (SD)	61.9 (9.3)	61.6 (9.7)	61.5 (9.0)	2	0.26^c^	.77
Range	40-82	37-88	39-87	2	0.44^d^	.80
Female, No. (%)	212 (57.8)	915 (52.4)	302 (55.7)	2	4.55^e^	.10
Education, mean (SD), y	14.1 (2.8)	14.1 (2.6)	14.1 (2.6)	2	0.10^c^	.90
Cardiovascular diseases, No. (%)	40 (10.9)	208 (11.9)	74 (13.7)	2	1.78^e^	.41
Follow-up after CRP measurement, mean (SD), y	14.5 (3.9)	14.3 (3.9)	14.3 (4.0)	2	0.37^c^	.69
**Examination 2**						
CRP, median (IQR), mg/L	1.1 (0.5-2.6)	1.0 (0.4-2.4)	0.8 (0.3-2.0)	2	22.25^d^	<.001
CRP ≥3 mg/L, No. (%)	79 (21.5)	351 (20.1)	84 (15.5)	2	6.88^e^	.03
**Examination 6**^f^						
CRP, median (IQR), mg/L	2.1 (0.9-5.0)^g^	2.2 (1.0-5.0)^g^	1.8 (0.8-4.0)^g^	2	14.61^d^	<.001
CRP ≥3 mg/L, No./Total No. (%)	136/339 (40.1)	661/1635 (40.4)	163/499 (32.7)	2	7.10^e^	.03
**Examination 7**^f^						
CRP, median (IQR), mg/L	2.6 (1.2-5.7)^g^	2.3 (1.1-5.2)^g^	1.8 (0.9-4.3)^g^	2	20.97^d^	<.001
CRP ≥3 mg/L, No./Total No. (%)	156/340 (45.9)	700/1633 (42.9)	180/501 (35.9)	2	10.18^e^	.006
CRP levels greater than or equal to the cutoff value in 2 measurements						
CRP ≥3 mg/L, No. (%)	122 (33.2)	573 (32.8)	132 (24.4)	2	14.64^e^	.001
Age for the subgroup, mean (SD), y	63.7 (9.1)	63.2 (9.1)	64.2 (8.3)	2	0.66^c^	.51
CRP ≥8 mg/L, No. (%)	42 (11.4)	132 (7.6)	27 (5.0)	2	13.07^e^	.001
Age for the subgroup, mean (SD), y	62.5 (9.0)	62.8 (9.2)	66.6 (8.9)	2	2.07^c^	.13
Outcomes, No. (%)						
Mortality	92 (25.1)	487 (27.9)	151 (27.9)	2	1.25^e^	.54
Cognitive outcomes						
Incident cases of dementia	21 (5.7)	105 (6.0)	65 (12.0)	2	21.04^e^	<.001
Incident cases of AD	14 (3.8)	83 (4.8)	55 (10.1)	2	22.20^e^	<.001

Abbreviations: AD, Alzheimer disease; CRP, C-reactive protein; IQR, interquartile range.

SI conversion factor: To convert CRP to nanomoles per liter, multiply by 9.524.

^a^ Mean (SD) with 1-way analysis of variance was used to test differences in CRP level and other variables among *ApoE* subgroups; median (IQR) with Kruskal-Wallis test with a χ^2^ value was applied when a concentration distribution was skewed. χ^2^ test was used to compare counts, No./Total (%).

^b^ *P* values for statistical significance are shown for *ApoE2*, *ApoE3*, and *ApoE4* comparisons.

^c^ *F* value.

^d^ χ^2^ Value in Kruskal-Wallis test.

^e^ χ^2^ Value in χ^2^ test.

^f^ Mann-Whitney test was for comparisons of CRP level at either examination 6 or 7 with CRP levels at examination 2 within each *ApoE* subgroup.

^g^ *P* < .05.

There was a positive association between increasing mean (SD) age and higher median (interquartile range) CRP levels (43.3 [9.6] years, 0.95 mg/L [0.40-2.35 mg/L] vs 59.1 [9.6] years, 2.04 mg/L [0.93-4.75 mg/L] vs 61.6 [9.5] years, 2.21 mg/L [1.05-5.12 mg/L]; *P* < .001) and a higher proportion of older participants had CRP levels of 3 mg/L or higher across all *ApoE* genotypes (Table 1). *ApoE4* carriers consistently had lower levels of CRP than did *ApoE2* and *ApoE3* carriers at each of the examinations. Overall, age was positively associated with chronic low-grade systemic inflammation, while *ApoE4* was negatively associated (Table 1).

### Association of the Interaction of *ApoE4* and Chronic Low-Grade Inflammation With Increased Risk of AD

**Figure 1.**
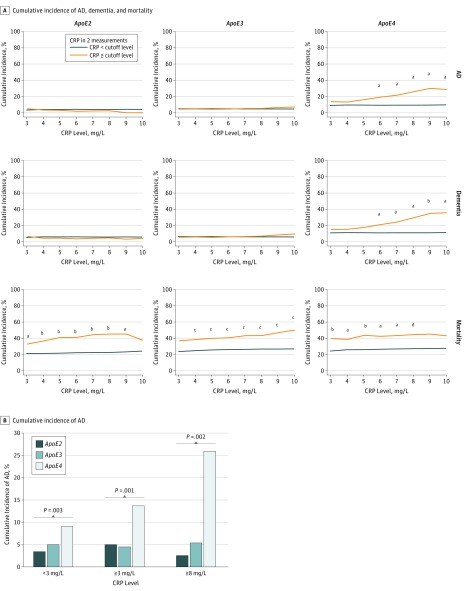
Cumulative Rates of Dementia, Alzheimer Disease (AD), and Mortality Based on *ApoE* Alleles and Chronic Low-grade Inflammation A, Cumulative incidence of AD, dementia, and mortality. Individuals were divided into *ApoE2*, *ApoE3*, and *ApoE4* genotypes. To define the absence and presence of chronic low-grade inflammation, C-reactive protein (CRP) cutoff levels at 2 measurement points are used to represent severity. The incident rates of AD, dementia, and mortality between those without and with different severity levels of chronic low-grade inflammation were compared by using the χ^2^ test. To convert CRP to nanomoles per liter, multiply by 9.524. ^a^*P* < .05. ^b^*P* < .01. ^c^*P* < .001. ^d^*P* = .08. B, Cumulative incidence of AD. Individuals were divided into *ApoE2, ApoE3*, and *ApoE4* genotypes for CRP concentrations of 3 mg/L or lower, 3 mg/L or higher, and 8 mg/L or higher. The AD incident rates were compared among the 3 *ApoE* subgroups by using the χ^2^ test for each level of CRP concentration.

The mean (SD) follow-up from the last CRP measurement was approximately 14.1 (4.2) years and there were no significant differences in follow-up time among the 3 *ApoE* groups (Table 1). Of the 194 individuals (7.3%) who developed dementia, 152 (78.4%) were diagnosed with AD. Using χ^2^ analyses, we tested whether the association between *ApoE* genotype and chronic low-grade inflammation status with the risk for incident dementia including AD and mortality changed when using different CRP cutoff levels (Figure 1). Those with both the *ApoE4* allele and chronic low-grade inflammation demonstrated a CRP level–dependent pattern that was linked to increased risk of AD and dementia (Figure 1A). This phenomenon was not observed in *ApoE2* and *ApoE3* carriers. *ApoE4* carriers with CRP levels of 3 mg/L or less had a onefold to twofold increase of risk of AD compared with *ApoE3* and *ApoE2* carriers with similar CRP levels (37 of 410 [9.0%] vs 58 of 1174 [4.9%] vs 8 of 245 [3.3%]; *P* = .003; Table 1 and Figure 1B). For CRP levels of 3 mg/L or higher, *ApoE4* carriers had a twofold to threefold increase of risk of AD compared with *ApoE3* and *ApoE2* carriers (18 of 132 [13.6%] vs 25 of 573 [4.4%] vs 6 of 122 [4.9%]; *P* = .001). For CRP levels of 8 mg/L or higher, *ApoE4* carriers had a fivefold to 10-fold increase of risk of AD risk compared with *ApoE3* and *ApoE2* carriers (7 of 27 [25.9%] vs 7 of 132 [5.3%] vs 1 of 42 [2.4%]; *P* = .002). In contrast, as expected, chronic low-grade inflammation was positively associated with or showed a positive trend with mortality rates across all *ApoE* genotypes (Figure 1).

We next used Cox proportional hazards regression analyses, adjusted for age, sex, educational level, cardiovascular disease, and *ApoE4* status. Although chronic low-grade inflammation, based on a CRP level of 8 mg/L or higher, 9 mg/L or higher, or 10 mg/L or higher, was not found to be associated with AD or dementia, the interaction between *ApoE4* and chronic low-grade inflammation was associated with AD or dementia (CRP level ≥9 mg/L: hazard ratio, 3.47; 95% CI, 1.10-10.94; *P* = .03) or tended to be positively associated with AD. To determine if cardiovascular diseases may account for this association, we excluded individuals with cardiovascular diseases and found that, in the absence of cardiovascular diseases, the association was significantly stronger (hazard ratio, 6.89; 95% CI, 1.74-27.30; *P* = .006) (Table 2). In addition, after stratifying participants by *ApoE4* status, we found that chronic low-grade inflammation was significantly associated with AD risk only in *ApoE4* carriers (hazard ratio, 4.70; 95% CI, 1.83-12.04; *P* = .001), but not in *ApoE2* or *ApoE3* carriers. Again, chronic low-grade inflammation was positively associated with mortality across all *ApoE* genotypes, but the interaction between *ApoE4* and chronic low-grade inflammation was not associated with mortality (Table 2).

**Table 2.** Cox Proportional Hazards Regression Models for the Risk of Chronic Low-grade Inflammation on the Incidence of Dementia, Alzheimer Disease, and Mortality

CRP Cutoff Level^a^	No. (%)	Model^b^	Alzheimer Disease		Dementia		Mortality	
			HR (95% CI)	*P* Value	HR (95% CI)	*P* Value	HR (95% CI)	*P* Value
**All Participants (N = 2656 [100.0%])**								
8 mg/L	201 (7.6)	1	1.22 (0.71-2.10)	.47	1.33 (0.82-2.15)	.25	1.78 (1.42-2.23)	<.001
With *ApoE4*		2	2.64 (0.89-7.80)	.08	2.44 (0.92-6.45)	.07	0.81 (0.43-1.53)	.52
9 mg/L	160 (6.0)	1	1.43 (0.80-2.55)	.22	1.59 (0.96-2.64)	.07	1.88 (1.47-2.41)	<.001
With *ApoE4*		2	3.47 (1.10-10.94)	.03	2.98 (1.08-8.27)	.04	0.84 (0.41-1.74)	.65
10 mg/L	122 (4.6)	1	1.39 (0.73-2.66)	.32	1.66 (0.96-2.89)	.07	1.79 (1.36-2.35)	<.001
With *ApoE4*		2	2.76 (0.74-10.30)	.13	2.46 (0.87-7.71)	.12	0.77 (0.32-1.83)	.55
**Individuals Without CVD (n = 2334 [87.9%])**								
8 mg/L	158 (6.8)	1	1.23 (0.64-2.37)	.54	1.33 (0.75-2.37)	.33	1.71 (1.28-2.27)	<.001
With *ApoE4*		2	6.63 (1.80-24.5)	.005	4.14 (1.28-13.44)	.02	0.80 (0.31-2.07)	.65
9 mg/L	125 (5.4)	1	1.42 (0.72-2.83)	.31	1.60 (0.88-2.92)	.12	1.86 (1.36-2.54)	<.001
With *ApoE4*		2	6.89 (1.74-27.30)	.006	4.07 (1.22-13.59)	.02	0.88 (0.34-2.29)	.80
10 mg/L	95 (4.1)	1	1.21 (0.53-2.78)	.65	1.56 (0.79-3.09)	.20	1.78 (1.24-2.54)	.002
With *ApoE4*		2	6.82 (1.31-35.6)	.02	3.61 (0.86-15.16)	.08	0.99 (0.30-3.30)	.98

Abbreviations: CRP, C-reactive protein; CVD, cardiovascular diseases; HR, hazard ratio.

^a^ The CRP levels greater than or equal to a given cutoff at least in 2 measurements to define chronic low-grade inflammation.

^b^ Model 1: HR of chronic low-grade inflammation after adjusting for age, sex, educational level, *ApoE*, and CVD. Model 2: HR of the interaction effects between chronic low-grade inflammation and *ApoE4*, adjusting for all covariates in model 1.

### Interaction of *ApoE4* and Chronic Low-grade Inflammation and Latency of Onset of AD

**Figure 2.**
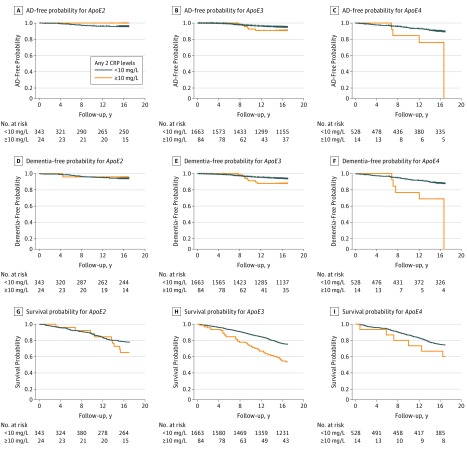
Kaplan-Meier Analysis for Survival Free of Alzheimer Disease (AD), Dementia, and Mortality in the Context of *ApoE* Alleles and Chronic Low-grade Inflammation A, AD-free probability for *ApoE2 *(*P* = .32). B, AD-free probability for *ApoE3 *(*P* = .14). C, AD-free probability for *ApoE4 *(*P* = .009). D, Dementia-free probability for *ApoE2 *(*P* = .74). E, Dementia-free probability for *ApoE3 *(*P* = .06). F, Dementia-free probability for *ApoE4 *(*P* = .001). G, Survival probability for *ApoE2 *(*P* = .18). H, Survival probability for *ApoE3 *(*P* < .001). I, Survival probability for *ApoE4 *(*P* = .19). C-reactive protein (CRP) cutoff level of 10 mg/L or higher at a minimum of 2 time points was used to define chronic low-grade inflammation.

Using Kaplan-Meier analysis, we found that individuals with *ApoE4* and chronic low-grade inflammation, defined as having a CRP cutoff level of 10 mg/L or more in at least 2 examinations, was more strongly associated with onset of dementia as well as AD compared with *ApoE4* carriers without this level of inflammation. In comparison, chronic low-grade inflammation status did not affect risk of dementia including AD in *ApoE2* carriers. Results for *ApoE3* carriers fell between findings for *ApoE2* and *ApoE4* carriers, showing marginal influences of chronic low-grade inflammation on risk of AD. We also tested cutoff CRP levels of 8 mg/L or higher and 9 mg/L or higher to examine severity of chronic low-grade inflammation and found that they were all linked to onset of AD in the Kaplan-Meier analysis for carriers of *ApoE4*, but not for carriers of *ApoE2* and *ApoE3* (eFigure 2 in the Supplement). Again, chronic low-grade inflammation was significantly associated with a low survival rate in *ApoE3* carriers and showed this same trend in *ApoE2* and *ApoE4* carriers (Figure 2).

### Association of the Interaction of *ApoE4* and Chronic Low-grade Inflammation With Brain Atrophy

The above findings suggest that chronic systemic inflammation may make the brain more vulnerable to AD. To test this hypothesis, we used brain volumetric measures acquired after the last CRP measurement from 1761 participants. The mean (SD) time from the first CRP measurement to the brain MRI scan was 24.0 (3.1) years, mean (SD) time from the second CRP measurement to the brain MRI scan was 8.5 (3.1) years, and mean (SD) time from the third CRP measurement to the brain MRI scan was 5.6 (3.0) years. The brain volumes of those without (n = 1647) and with (n = 114) chronic low-grade inflammation, defined by cutoff CRP levels of 8 mg/L or higher at a minimum of 2 examinations, were compared (eTable in the Supplement). With 1 exception, we found no differences in brain regions between those without and with chronic low-grade inflammation after Bonferroni corrections. There was a significant difference between the 2 groups for total white matter volume. These associations held for multivariate linear regressions that adjusted for age, sex, educational level, *ApoE4* status, and the time between CRP and brain MRI measures (Table 3). The interaction of *ApoE4* and chronic low-grade inflammation, however, was negatively associated with the regions associated with AD pathologic characteristics (eg, temporal lobe brain volume: β = –0.78, SE = 0.25; *P* = .01) and persisted after adjusting for age and sex (temporal lobe brain volume: β = –0.85, SE = 0.22; *P* < .001) as well as after adjusting for age, sex, time to brain MRI, educational level, and cardiovascular disease (temporal lobe brain volume: β = –0.88, SE = 0.22; *P* < .001). This same association was found for hippocampal volume in the model adjusted for age, sex, time to brain MRI, educational level, and cardiovascular disease (β = –0.04, SE = 0.01; *P* = .005). No other significant associations were found for other brain regions.

**Table 3.** General Linear Regression Analyses of Chronic Low-grade Inflammation Effect and the Interaction Effect Between *ApoE4* and Chronic Low-grade Inflammation on Brain Volumes

Outcomes (n = 1785)	Model^a^	Chronic Low-grade Inflammation Alone, High CRP Level (≥8 mg/L Twice)		Interaction Effect, *ApoE4* × High CRP Level (≥8 mg/L Twice)	
		β Estimate (SE)	*P* Value^b^	β Estimate (SE)	*P* Value^b^
TCBV	1	−18.06 (12.97)	.82	−10.16 (35.59)	>.99
	2	−14.07 (12.92)	>.99	−16.52 (35.45)	>.99
FBV/TCBV%^c^	1	−0.20 (0.13)	.60	−0.08 (0.35)	>.99
	2	−0.19 (0.13)	.74	−0.13 (0.35)	>.99
PBV/TCBV%^c^	1	−0.03 (0.08)	>.99	−0.42 (0.25)	.10
	2	−0.05 (0.08)	>.99	−0.52 (0.24)	.10
TBV/TCBV%^c^	1	−0.10 (0.08)	>.99	−0.85 (0.20)	<.001
	2	−0.09 (0.09)	>.99	−0.88 (0.22)	<.001
HPV/TCBV%^c^	1	−0.004 (0.005)	>.99	−0.04 (0.02)	.01
	2	−0.004 (0.005)	>.99	−0.04 (0.02)	.01

Abbreviations: CRP, C-reactive protein; FBV, frontal lobe brain volume; HPV, hippocampal volume; PBV, parietal lobe brain volume; TBV, temporal lobe brain volume; TCBV, total cerebral brain volume.

^a^Model 1: adjusted for age and sex. Model 2: model 1 plus time to magnetic resonance imaging, educational level, and *ApoE4*.

^b^With Bonferroni correction.

^c^Percentage of TCBV indicates brain atrophy.

## Discussion

C-reactive protein is a biomarker of low-grade inflammation. To our knowledge, this is the first study to use longitudinal measurements of CRP to define a chronic condition of low-grade inflammation at baseline and demonstrate that *ApoE4* interacting with chronic low-grade inflammation increased the risk of AD and shortened the latency for developing AD (Figure 1 and Figure 2). Since it is well documented that infection and inflammation are common in elderly individuals, and preclinical studies have reported that inflammation induced AD pathologic characteristics in mice who only carried *ApoE4*,^16,17^ our findings may explain why *ApoE4* carriers have increased risk for AD at an old age and suggest that treating chronic low-grade inflammation may delay the onset of AD in *ApoE4* carriers.

The strength of this study was its longitudinal follow-up for incident cases of dementia that was preceded by multiple measurements of CRP with an interval between CRP measurements of 6 to 16 years (Table 1). This study offsets the limitation of earlier studies that relied on onetime measurement of CRP to study the development of AD that has resulted in reports of positive,^5^ negative,^6,18^ and no association^7,19,20,21^ between CRP and AD. Although these studies cannot distinguish between those who had an elevated CRP level and then recovered vs those who had a sustained or multiple episodically elevated CRP levels, based on our data analyses we hypothesized that genetic vulnerability for AD might be associated with a long-term low-grade inflammatory condition, albeit either sustained or episodic.

Although genetic risk factors such as *ApoE4* for AD are present across the lifespan, disease onset does not occur until later in life.^2^ Our findings suggest that chronic low-grade inflammation interacts with *ApoE4* to accelerate the onset of AD in a pattern dependent on the CRP level (Figure 1 and Figure 2; eFigure 2 in the Supplement). Although *ApoE2* carriers had higher levels of CRP with increasing age than did *ApoE3* and *ApoE4* carriers (Table 1), high CRP levels were not associated with risk of AD among *ApoE2* carriers (Figure 1 and Figure 2). It is probable that chronic low-grade inflammation linked with *ApoE4* puts the brain into a vulnerable state for the development of AD (Table 3), but the brain of *ApoE2* carriers is resilient to the influence of chronic low-grade inflammation on the development of AD. This possibility is consistent with other studies that report other proinflammatory factors associated with brain atrophy.^22,23^ Another study found that periodontal disease, another common inflammatory condition in elderly individuals, is associated with a higher brain amyloid load detected by amyloid positron emission tomographic scan in healthy elderly individuals.^24^ Together, these studies suggest a possible link between systemic infection and AD pathologic characteristics in the brain in humans. We propose that if chronic low-grade inflammation is detected through follow-up CRP measurements and is treated among elderly individuals who are *ApoE4* carriers, the onset of AD can be delayed or even prevented, since studies have reported that delaying onset by 5 years can reduce the risk for AD by nearly 50%.^25^

The association between chronic low-grade inflammation and risk of AD for *ApoE4* carriers became even more significant in the absence of cardiovascular diseases (Table 2). Since CRP levels are linked to cardiovascular disease,^26^ which is also a risk factor for AD, our results indicate that chronic low-grade inflammation may play an early role leading to AD in *ApoE4* carriers^4,8^ independent from cardiovascular diseases. Acute inflammatory reaction to infection or injury is a physiological process that is a defense mechanism of the body and is marked by an elevation of CRP levels; however, chronic low-grade inflammation is a pathologic process that may lead to chronic diseases such as cardiovascular disease.^26^ Although chronic low-grade inflammation was linked to high rates of mortality across all *ApoE* genotypes, an increased risk of AD was found only in *ApoE4* carriers (Table 2, Figure 1, and Figure 2).

The mechanism for the interaction between *ApoE4* and a high sustained level of CRP that leads to an increased risk of AD is unknown. Both *ApoE* and CRP are produced mainly by the liver, implying a liver-brain inflammation axis for the pathogenesis of AD. A liver-brain inflammation axis has been proposed to cause abnormal clinical symptoms in brain diseases, including mood diseases, cognition diseases, and neurovegetative signs,^27^ but its association with AD remains unclear. Gram-negative bacteria often cause infection in elderly individuals, including in the gastrointestinal, respiratory, and urinary systems. Injection of the gram-negative bacterial cell wall component lipopolysaccharides can increase levels of CRP in the blood,^28,29,30^ implying that the level of CRP could be a biomarker after attacks from bacteria endotoxin lipopolysaccharides. Systemic administration of lipopolysaccharides into AD mouse models induced AD pathologic characteristics in the brain.^16^ It has been shown that lipopolysaccharide challenge is linked to cerebrovascular pathologic findings and increased amyloid burden in an *ApoE4* AD mouse model, but not in a non-*ApoE4* AD mouse model.^17^ One recent study found that the amount of lipopolysaccharides in the brains of humans with AD are twofold higher compared with control brains.^31^

### Limitations

Although a strength of this study is the longitudinal measurements of CRP, a limitation is that there were not more frequent, preferably annual, CRP measures; thus, it is possible that some cases of sustained inflammatory status may have been misclassified or missed. It is also possible that we have underestimated the interactive association of *ApoE4* and chronic low-grade inflammation with AD. Furthermore, the Framingham Heart Study cohort lacks ethnic diversity and thus these findings lack generalizability to nonwhite populations.

## Conclusions

As systemic infection and inflammatory attacks are common in elderly individuals, recovery of the immune system to baseline could be critical for certain genotypes such as *ApoE4* for the sequela of AD development. Evidence of chronic low-grade inflammation stage could be targeted for personalized treatment. Our findings provide initial evidence of the importance of *ApoE* genotype in clinical trial studies of anti-inflammatory drugs for AD. Although previous clinical trials of anti-inflammatory drugs for AD have failed,^32^ specifically targeting a subset of patients based on *ApoE* genotypes and inflammation status may be an important consideration for future clinical trial study design. Additional studies are warranted to determine whether rigorous treatment of infection and inflammation that lower CRP levels to a normal level will attenuate the risk of AD for *ApoE4* carriers.

## Notes

Supplement. **eTable.** Brain Volume Comparisions Between Those With Low vs High Chronic Low-grade Inflammation: CRP ≥ 8 mg/dL at a Minimum of 2 Time Points **eFigure 1.** Sample Selection Flowchart **eFigure 2.** Kaplan-Meier Analysis for AD Dementia-Free Survival in the context of *ApoE* Alleles and Chronic Low-grade Inflammation
