# Supplementary material for: Association of Chronic Low-grade Inflammation With Risk of Alzheimer Disease in ApoE4 Carriers
Source: JAMA Netw Open. 2018 Oct 19;1(6):e183597. doi: 10.1001/jamanetworkopen.2018.3597 (PMC6324596; doi:10.1001/jamanetworkopen.2018.3597)
Supplement: Supplement. — eTable. Brain Volume Comparisions Between Those With Low vs High Chronic Low-grade Inflammation: CRP ≥ 8 mg/dL at a Minimum of 2 Time Points eFigure 1. Sample Selection Flowchart eFigure 2. Kaplan-Meier Analysis for AD Dementia-Free Survival in the context of ApoE Alleles and Chronic Low-grade Inflammation [file jamanetwopen-1-e183597-s001.pdf]

Supplementary Online Content

Tao Q, Ang TFA, DeCarli C, et al. Association of chronic low-grade inflammation with risk of Alzheimer disease in *ApoE4* carriers. *JAMA Netw Open.* 2018;1(6):e183597. doi:10.1001/jamanetworkopen.2018.3597

eTable. Brain Volume Comparisions Between Those With Low vs High Chronic Low-grade Inflammation: CRP ≥ 8 mg/dL at a Minimum of 2 Time Points
eFigure 1. Sample Selection Flowchart
eFigure 2. Kaplan-Meier Analysis for AD Dementia-Free Survival in the context of *ApoE* Alleles and Chronic Low-grade Inflammation

This supplementary material has been provided by the authors to give readers additional information about their work.

© 2018 Tao Q et al. *JAMA Network Open.*

eTable. Brain Volume Comparisions Between Those With Low vs High Chronic Low-grade Inflammation: CRP ≥ 8 mg/dL at a Minimum of 2 Time Points

**Brain volumes, Mean ± SD**	**All subjects who had a brain MRI**		***P* values**
	Without CRP ≥ 8 mg/L twice (*n* = 1647)	With CRP ≥ 8 mg/L twice (*n* = 114)	
**Age at Exam 7,** years	60.9 ± 9.3	62.7 ± 9.5	.05
**Females,** n (%)	866 (52.6)	78 (68.4)	.001
**TCBV,** cm^3	1415.5 ± 140.3	1369.6 ± 144.2	.001
**Gray/TCBV %**	41.3 ± 1.8	41.5 ± 2.1	.50
**White/TCBV %**	34.4 ± 2.4	34.1 ± 2.8	.16
**FBV**			
**Gray/TCBV %**	12.0 ± 0.8	11.9 ± 0.8	.46
**White/TCBV %**	11.7 ± 1.0	11.6 ± 11.0	1.00
**PBV**			
**Gray/TCBV %**	6.7 ± 0.4	6.6 ± 0.5	.52
**White/TCBV %**	6.3 ± 0.5	6.3 ± 0.6	.85
**TBV**			
**Gray/TCBV %**	8.4 ± 0.5	8.4 ± 0.6	.59
**White/TCBV %**	5.3 ± 0.5	5.1 ± 0.5	.01 (.05) *
**HPV/TCBV %**	0.47 ± 0.04	0.47 ± 0.04	.69
**Log(WMHI)/TCBV %**	0.006 ± 0.08	0.003 ± 0.09	.48

1761 subjects who had a brain MRI were included. Chronic low-grade inflammation was defined as having CRP ≥ 8 mg/L at a minimum of two time-points. Mean ± SD with t-tests with *P* values are presented to compare brain volumes between those with low vs. high chronic low-grade inflammation status. TCBV = total cerebral brain volume; FBV = frontal lobe brain volume; TBV = temporal lobe brain volume; HPV = hippocampal volume; Log(WMHI) = Logarithm transformation of white matter hyperintensities volume. The ratios of each brain region over TCBV were compared. P values for statistical significance are shown. **P* value after Bonferroni correction

© 2018 Tao Q et al. *JAMA Network Open.*

eFigure 1. Sample Selection Flowchart

A). The source population is 3130 participants, who were aged 20 years and above at the 2nd health exam at which baseline CRP was measured and known ApoE genotype. The exclusion criteria for the primary analysis are:

a) No serial CRP measurement at 6th and/or 7th health exams (n = 404)
b) ApoE 2/4 (n=46)
c) Prevalent dementia (n = 24)

895 participants were excluded from the secondary analysis due to the absence of a brain MRI after the last longitudinal measure of CRP.

B). The study design illustrated shows the year durations between the three longitudinal CRP measures and follow-up for incident cases of dementia/AD.

Supplement Figure 1A

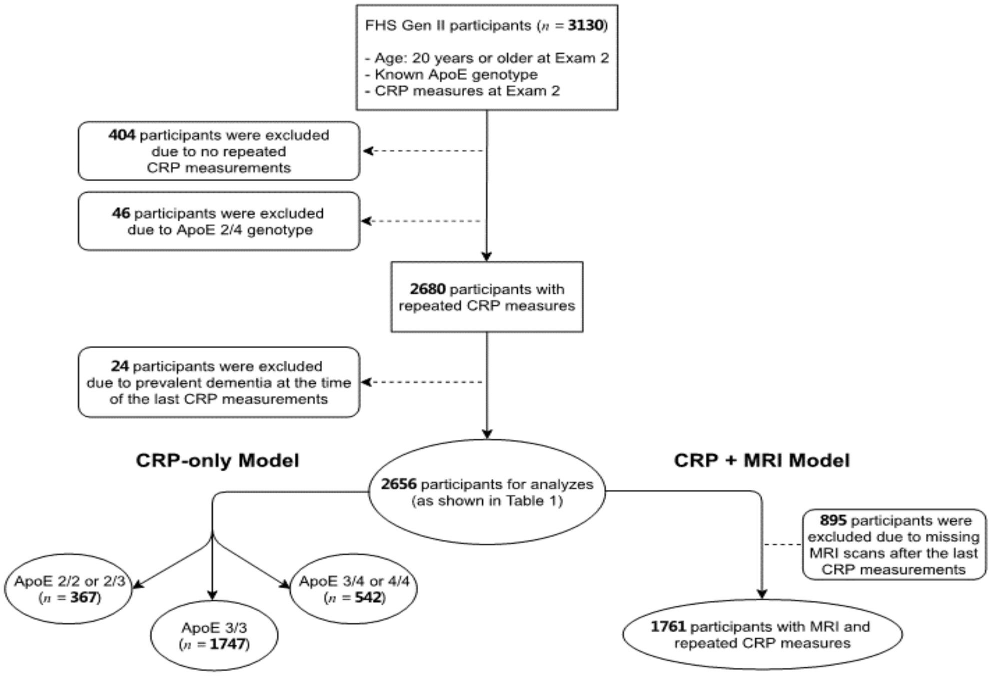

© 2018 Tao Q et al. *JAMA Network Open.*

Supplement Figure 1B

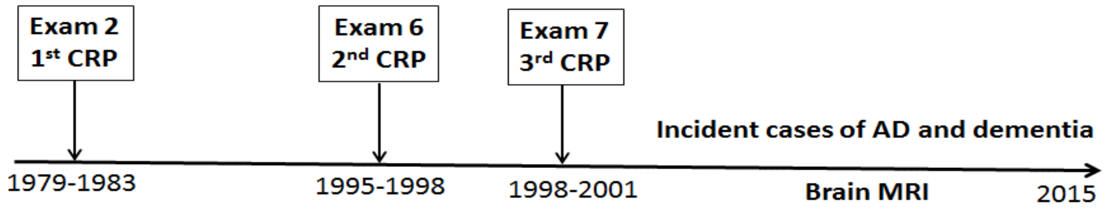

© 2018 Tao Q et al. *JAMA Network Open.*

eFigure 2. Kaplan-Meier Analysis for AD Dementia-Free Survival in the context of *ApoE* Alleles and Chronic Low-grade Inflammation

Subjects were divided into ApoE2, ApoE3 and ApoE4 genotype. CRP cutoff levels, < and ≥ 8 mg/L, < and ≥ 9 mg/L and < and ≥ 10 mg/L at a minimum of two time-points were used to define the absence and presence of chronic low-grade inflammation. Kaplan-Meier survival analysis was used to study the survival dementia free time before AD onset during 17 years of follow-up. The survival curves (A) and the numbers of each group at each follow-up point (B) are shown.

Supplement Figure 2

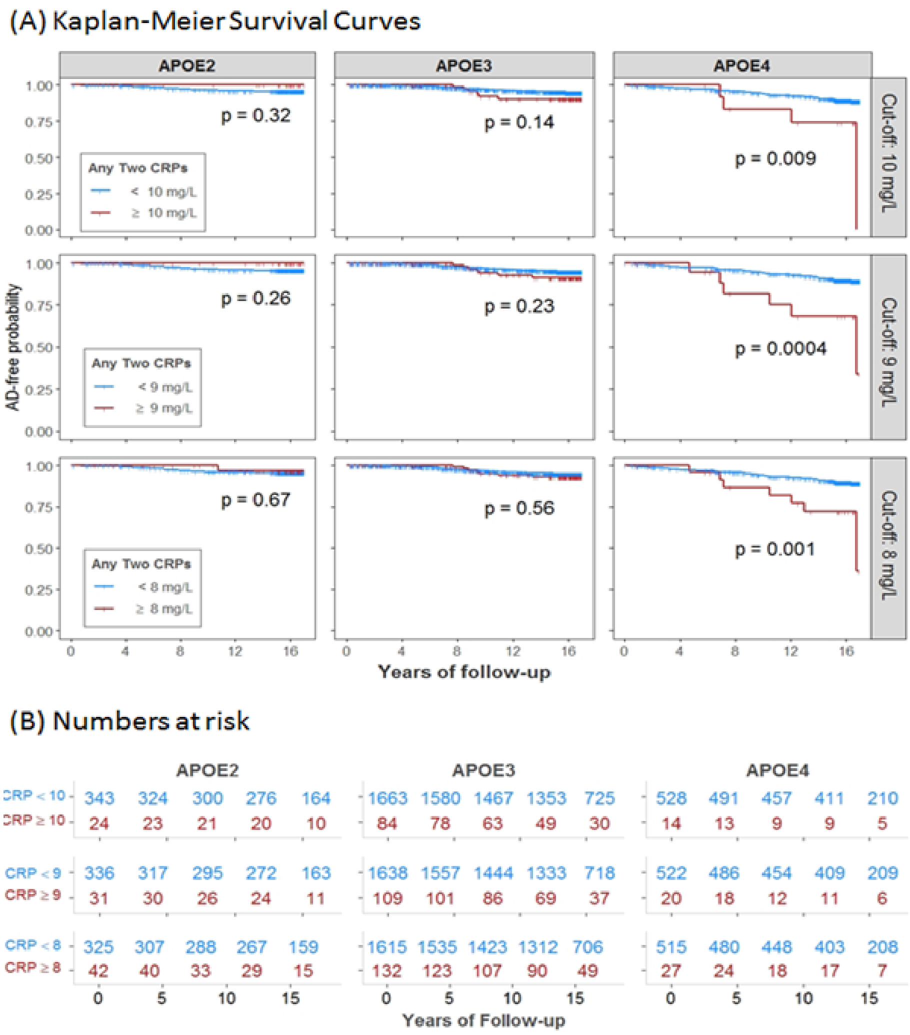

© 2018 Tao Q et al. *JAMA Network Open.*
